# Short-Term Attentional Perseveration Associated with Real-Life Creative Achievement

**DOI:** 10.3389/fpsyg.2013.00191

**Published:** 2013-04-25

**Authors:** Darya L. Zabelina, Mark Beeman

**Affiliations:** ^1^Department of Psychology, Northwestern UniversityEvanston, IL, USA

**Keywords:** creative achievement, divergent thinking, attentional flexibility, cognitive flexibility, persistence, creativity, perseveration

## Abstract

There are at least two competing hypotheses of how attention interacts with creative cognition, although they are not mutually exclusive. The first hypothesis is that highly creative people are particularly flexible at switching their attention – that is, they adaptively shift focus among different attentional levels using cognitive control. The second, less common, view is that creative people exhibit attentional persistence, or an ability for sustained attention. We suggest these two views need not be competing, as they may both operate, but on different time scales or on different components of creativity. In the present study we examined the role of attention in real-world creative achievement and in divergent thinking. In Experiment 1 participants with high and low real-world creative achievements identified whether the stimulus contained letters S or H within hierarchically constructed letters (e.g., large S made of small Es – global level; large E made up of small Ss – local level), which were presented in blocks of eight trials per level. In Experiment 2 participants with high, medium, and low creative achievements identified the same stimulus letters, but in blocks of five, seven, and nine trials per level. Results from both experiments indicated that people with high creative achievements made significantly more errors on trials in which they had to switch the level of attention, even after controlling for general intelligence. In Experiment 2, divergent thinking was also assessed, but it was not related to switching cost. Results from both experiments demonstrate that real-world creative acts relate to increased levels of attentional persistence, even if it comes with the cost of perseveration in certain circumstances.

## Introduction

Creativity is thought to capitalize on a distinct form of attention, yet how attention differs in creative people remains unresolved. There are at least two seemingly contradictory hypotheses about how attention relates to creative cognition. The first is a common view that creative people have the *attentional flexibility* to adaptively shift focus between the two types of attentional foci using cognitive control (e.g., Stavridou and Furnham, 1996; Vartanian, 2009). A second view is that creative people have the *attentional persistence* to focus for extended durations, which is fundamental to creative production (Groborz and Neçka, 2003). These two hypotheses need not be competing. Rather, they may both operate, but on different components of creativity, with two different factors of attention at work. Specifically, cognitive flexibility may be more descriptive of divergent thinking – the ability to generate many diverse ideas, while attentional persistence may be required for real-world creative achievements.

### Divergent thinking and attentional flexibility

One component of creativity is divergent thinking, which is measured by tasks such as the Torrance Test of Creative Thinking (TTCT; Torrance, 1974). According to the attentional flexibility hypothesis, creative acts require the ability for rapid and fluid switching between different types of foci – switching between various ideas/stimuli, as well as the ability to see the large picture and to pay attention to its details, and to flexibly switch between these two types of attention (Martindale, 1995; Gabora, 2010). Spontaneous shifts between analytic and associative modes of thought, for example, are suggested to be necessary for creative production (Gabora, 2010). Creative people are suggested to adjust their focus of attention depending on the task demands. Specifically, people with higher (compared to those with lower) creative potential, measured mostly by divergent thinking tests, are faster on tasks not involving interference or ambiguity, but are slower on tasks involving interference or ambiguity (Vartanian et al., 2007). These results suggest that people with high creative potential may in fact be more flexible along the attention continuum, speeding up when they can and slowing down when they must.

Attentional flexibility requires people to switch quickly between foci, i.e., between similar stimuli; or switch between levels of attention (broad or global versus narrow or local), or between degree of filtering (high filtering with suppression of stimuli outside attention versus low filtering). It is also unclear what timescale of flexibility is required for successful real-world creative production. Rapid flexible attentional switching may be important for creativity in the short-term, such as for performance on timed tasks of insight problem-solving (e.g., Bowden and Beeman, 1998) or divergent thinking (e.g., Torrance, 1974). In the divergent thinking tasks used to measure creative potential, for example, people are given 3 min to name as many uses for a familiar object, such as a brick, as possible. Responses are scored for fluency (number of solutions), originality (novelty of solutions), and flexibility (the number of solution categories). In such tasks, rapid attentional switching is rewarded, particularly for the fluency and flexibility scores. Thus, it is likely that attentional flexibility will be related to these measures of divergent thinking, which is a component of creativity generally.

### Creative achievement and attentional persistence

There is less evidence for the view that creative people have persistent attention, particularly in the short-term – in part because there are fewer studies of attentional persistence, and in part because “creative people” is often defined as high divergent thinking scores. The Creative Achievement Questionnaire (CAQ) employed in this study (Carson et al., 2005) provides a self-report measure of how people have used creativity in the real-world (for details, see Methods).

Many or most real-world creative acts require some degree of persistence. Indeed, in order to create new ideas, new machines, or highly original paintings, people have to invest an inordinate amount of focus and persistence in the task at hand (Simonton, 1999). Since the time of Wallas (1926), *immersion*, i.e., extended preparation and thought, has been considered a critical stage of the creative process. In order to have a new idea, persistence on the topic is needed to learn what is already known in the field and what problems or issues could be addressed with new ideas. Others note that even when ideas come in a flash, persistence is required to put them to good use (Gabora, 2010). Deep thinking, for example, has been found to increase task shielding, and reduce shifting flexibility (Fischer and Hommel, 2012). According to the dual pathway to creativity model, creative originality can be reached through cognitive flexibility, as well as through exploring fewer categories in greater depth, i.e., through persistence (Nijstad et al., 2010). Persistence in the discovery process does not come easily. There are many conflicting demands, and creative ideas are often, by definition, incompletely formulated, or of dubious utility. What is the mechanism that allows creative achievers to persevere in the face of these difficulties?

Creative achievers may intrinsically be more likely to become engaged in tasks through sheer love of their pursuit, or through motivation. In addition, creative achievers may differ from less creative people in how thoughts initially capture their attention, and this may differ from attentional flexibility. Whatever the mechanism, eminent creative people are suggested to be virtually addicted to their work (Eysenck, 1995). Newton purportedly explained that he discovered the laws of gravity simply “by thinking on it continually” (Westfall, 1980). A considerable body of research does in fact suggest that creativity involves the ability to maintain an extended focus (Richards et al., 1988; Feist, 1999). Artists, for instance, spend more time re-working a drawing than do non-artists (Kozbelt, 2008). Thus, although some aspects of creativity can require some form of cognitive flexibility, the ability for persistence may be a defining characteristic of successful creative achievement.

How is persistence in the real-world related to persistence in a simple task of attention switching? It is possible that real-world creative achievement reflects personal characteristics, such as motivation, grit, or persistence. But it may also reflect more basic cognitive processes, such as participants’ attention or cognitive control that favors persistent attention at a level of focus. This study examines whether the capture or control of attention in the short-term relates to long-term creative achievement. To control for general intelligence, we took into account participants’ SAT and ACT scores.

### Present study and hypotheses

We conducted two experiments to examine how real-world creative achievers and divergent thinkers allot their attention, and how they switch their attention over time. In both experiments, participants identified target stimuli that could occur as either local features or as global configurations of features. Critically, unlike prior studies using randomly ordered stimuli (e.g., Vartanian et al., 2007), the stimuli in our experiments were presented in sets that repeated the level at which the target appeared – sets of eight global, followed by eight local, etc., in Experiment 1, and sets of five, seven, or nine repetitions of each level in Experiment 2. Although participants were not informed of this pattern, it implicitly encouraged them to maintain attention on one attentional level for some time before switching to the other level. Examining accuracy (and speed) of responses on trials in which the target level switched allowed us to examine individual differences in the ability to switch attention from one level to the other, as well as possible preferences for either the local or global level. We compared participants on the basis of real-world creative achievement, such as producing artwork or publishing a scientific article (Carson et al., 2005), and, separately, on a divergent thinking measure (Experiment 2; Goff and Torrance, 2002). The primary question was: once people establish an attentional set, are people with high creative achievements, or people with high divergent thinking scores, more or less able to switch attention to the other level? Specifically, if creative achievement is associated with attentional persistence, then in both Experiments high creative achievers should show cost when switching levels of attention, as evidenced by more errors or slower response times at the switch trials compared to all other trials within the task. Alternatively, if divergent thinking is associated with attentional flexibility, then in Experiment 2 high divergent thinkers should exhibit less cost when switching levels of attention.

## Experiment 1

### Method

#### Participants

Thirty-five undergraduate students (23 female, average age = 18.7 years) participated for course credit, after being selected from 261 students taking introduction to psychology class, who completed the CAQ (Carson et al., 2005; see below). Nineteen students with very low creative achievements (CAQ scores ranging 0–3) and 19 students with very high creative achievements (CAQ scores ranging 25–54) were initially selected. This was done in order to insure a full range of scores, given that random selection may not have yielded participants with very high creative achievements. Three participants from the low CAQ group did not show up for the experiment. Data from one person in the low creativity group was dropped from the analysis because his response times on the global-local attention task (Navon, 1977) were 3 SDs above the mean. Thus, data from 34 participants were included in further analyses: 19 with high and 15 with low creative achievement scores. Written consent was obtained from all participants, and the protocol was approved by the Northwestern University Institutional Review Board. Participants were tested individually in one 30-min session.

#### Materials and procedure

##### The creative achievement questionnaire

Creative behavior was assessed in terms of the Creative Achievement Questionnaire (CAQ: Carson et al., 2005) participants indicated their prior creative achievements in 10 creative domains (architectural design, creative writing, culinary arts, dance, humor, inventions, music, scientific discovery, theater and film, and visual arts). Questions in the Music domain, for example, range from “I have no training or recognized talent in this area (score of 0)” to “My compositions have been critiqued in a national publication (score of 7).” In the Scientific Discovery domain scores range from “I have no training or recognized ability in this field (score of 0)” to “My work has been cited by other scientists in national publications (score of 7).” Domain scores were then summed to form a single index of creative achievement. The CAQ has test-retest reliability of *r* = 0.81, internal consistency reliability of alpha = 0.96. It shows predictive validity against artist ratings of a creative product – a collage (*r* = 0.59, *p* < 0.01), and correlates with tests of creative potential, such as divergent thinking tests (*r* = 0.47, *p* < 0.01), the Creative Personality Scale (Gough, 1979; *r* = 0.33, *p* < 0.01), Intellect (Goldberg, 1992; *r* = 0.51, *p* < 0.01), and Openness to Experience (Costa and McCrae, 1992; *r* = 0.33, *p* < 0.01). In addition, CAQ shows discriminant validity with tests of IQ (Carson et al., 2005). Even though CAQ is a self-report measure, it has ecological validity in that it reflects actual creative behavior in the real-world.

##### Global-local task

The abilities to attend to local stimuli or global configurations, and to switch between them, were examined with a series of global letters (38 mm × 25 mm) composed of local letters (6 mm × 4 mm) (Navon, 1977). Stimuli were presented one at a time. On each trial, participants looked for a target letter – either an “S” or an “H” – that could occur at either the local or global level. Each vertical line making up a global letter was formed from five, and each horizontal line was formed from four, closely spaced local letters. It has been suggested that global processing takes precedence over local processing (e.g., Navon, 1981; Treisman, 1986); however, several studies have found that the global versus local advantage depends on the characteristics of the stimuli and task demands (e.g., Grice et al., 1983). The stimuli employed here were designed to be unbiased toward either level (see Bultitude et al., 2009; cf. Vartanian et al., 2007).

Participants viewed stimuli from a distance of approximately 60 cm from the computer display. On each trial, participants were first presented with a fixation cross (“+”) in the center of the screen for 1000 ms. Then, one of eight composite stimuli was randomly presented, and participants pressed an “S” key if the stimulus contained the letter S or an “H” key if the stimulus contained the letter H. They were asked to respond as quickly and accurately as possible. Four of the composite letters were global target letters composed of local distractors (an *S* made of *E*s, an *S* made of *A*s, an *H* made of *E*s, an *H* made of *A*s), and four were global distractors made from local target letters (an *E* made of *S*s, an *E* made of *H*s, an *A* made of *S*s, an *A* made of *H*s).

The key feature of the task was that the letters were presented in alternating blocks of eight local trials, followed by eight global trials, and so on, with the first block type (local or global) counterbalanced across participants. Such blocking of levels allowed for testing participants’ performance on the *switch trial*, i.e., the first trial in which the target occurred at a given level (local or global) versus the *non-switch trials*, i.e., all other trials in the task – the primary interest of the study. Overall, 64 local and 64 global trials were presented, in addition to 16 (unanalyzed) practice trials.

##### General intelligence

Participants provided their SAT and ACT scores, which were converted into percentile scores based on the national statistics for all test-takers in 2011 (*M* = 94.11, SD = 11.58).

### Results

Participants made an average of 2.73% errors (SD = 2.80). Because participants were selected for high versus low creative achievement, we performed group comparisons. Overall, people with high creative achievements made more errors (*M* = 3.58%, SD = 2.99) than those with low creative achievements (*M* = 1.67%, SD = 2.19), *t*(32) = −2.08, *p* < 0.05.

#### Switching and creative achievement

Our primary interest concerned whether people with high creative achievements make more or fewer errors when switching from one level of attention to the other. In other words, we were interested in whether creative achievement is related to switching costs. (Raw data for within block versus switching trials are in Table 1). Switching cost was calculated as the difference score, i.e., how many more errors participants made on switching trials compared to all other trials, i.e., switch trials minus all other trials. Switching cost was significantly higher for high creative achievers (*M* = 5.65%, SD = 9.41) compared to low creative achievers (*M* = 0.45%, SD = 2.88), *t*(32) = 2.06, *p* < 0.05. Thus, high creative achievers had initial difficulty switching from one attentional level to the other. However, this difficulty dissipated quickly (there was no difference by trial 2: high creative achievement group *M* = 2.96%, SD = 5.66; low creative achievement group *M* = 2.92%, SD = 6.63, *t* < 0.1).

**Table 1:** **Experiment 1: mean (and SD) percent error rates on switch trials versus all other trials for high and low creative achievers**.

	Creative achievement	
	High	Low
Switch trials (% errors)	8.53 (10.57)	2.07 (3.90)
Other trials (% errors)	2.88 (2.30)	1.61 (2.08)

A binary logistic regression indicated that high creative achievers had higher switching cost compared to low creative achievers even when controlling for general intelligence, *b* = 0.21, SE *b* = 0.11, *p* < 0.05.

#### Global versus local targets

Participants erred about equally often on global (*M* = 3.08%, SD = 3.52) and local trials (*M* = 2.39%, SD = 3.32), *t*(33) = 1.02, *p* = 0.31. Stimulus level (local versus global targets) did not reliably interact with creative achievement level, *F*(1, 32) = 0.21, *p* = 0.65. Furthermore, all participants made errors equally often when switching from global to local targets or vice versa, and this did not interact with creative achievement (all *F*s < 1.0).

#### Response time

We performed parallel analyses with response times as the dependent variable, excluding errors, and response times exceeding 3 SDs from the mean. There was no speed-accuracy tradeoff: high and low creative achievers showed equivalent switching costs in response time, even while high creative achievers showed a greater switching cost in accuracy. Overall, participants averaged 633 ms to respond to trials (SD = 94 ms). Participants responded equally quickly on global (*M* = 639 ms, SD = 99 ms) and local trials (*M* = 625 ms, SD = 100 ms), *t*(33) = 1.31, *p* = 0.20, which was expected given that our stimuli were designed not to have global precedence. Creative achievement groups did not differ on overall response time, and the type of trial (global versus local) did not interact with creative achievement level (low versus high), all *F*s < 1.0.

Predictably, participants responded more slowly on switch trials (*M* = 746 ms, SD = 156 ms) than on all other trials (*M* = 610 ms, SD = 95 ms), *F*(1, 32) = 55.75 *p* < 0.001. This again indicates that participants exhibited response time cost when switching from one attentional level to the other. However, this switching cost was roughly equal for high (141 ms) and low (134 ms) creative achievers, and switching did not interact with creative achievement (*F* < 1.0). Creative achievement groups did not differ in their response time cost in switching from global to local trials or vice versa (*t*s < 1.0).

## Discussion and Experiment 2

In Experiment 1, even when controlling for general intelligence, high creative achievers made more errors when switching levels of attention than did low creative achievers, while there were no differences in response times and no differences based on the type of stimulus (global versus local). Notably, high creative achievers performed worse overall on the task than did low creative achievers, in contrast to the intuition that people with high creative achievements would excel on most tasks. In particular, after a series of trials in which the targets appeared at the same attention level (either all global or all local), the high creative achievers had more difficulty switching to the other level than did participants with low creative achievement. This demonstrates a novel link between attentional persistence and creative achievement, a distinct effect from previous reports that creativity (at least, divergent thinking) is related to attentional flexibility (e.g., Vartanian et al., 2007). Although on the surface these results might seem to differ from previous findings, we suggest this is a distinct effect of attentional switching that results from a different mechanism of attention, and is not necessarily in opposition with prior claims.

It is possible, though not plausible, that high creative achievers were better at noticing the pattern of the blocked levels (so attended more to the block level and were thus more affected by the switch), whereas low creative achievers ignored the pattern. However, this explanation is unlikely, as both groups made more errors when switching target levels; moreover, high creative achievers actually made slightly more errors than low creative achievers even on non-switch trials, so they failed to show greater benefit within a run of trials at a level. Alternatively, perhaps *low* creative achievers not only noticed the sequences, but knew exactly when to switch their attention, and so showed less cost when switching between levels. However, the low creative achievers did show a cost (in both accuracy and response time) when switching levels of attention. Further, in debriefing, no participants reported noticing the target levels were blocked. It is also possible that high creative achievers were simply bored or distracted, and therefore were off task, however, error rates within the block were still very low, and, most importantly, there was a significant interaction between the switch trials and all other trials within the block. Given the nature of the task, it does not seem that being off task would predict errors specifically at the switch point. It is also important to note that participants who performed worse on the task were high creative achievers, and should be expected to be persistent on any task. A final concern is that the error rates for most trials, other than the switch trial, were so low that a floor effect may have masked group differences that occurred in trials 2–8, the non-switch trials.

To address these issues, we made several changes for Experiment 2: we decreased the predictability of the switch trials by varying the sequence length; and to eliminate potential floor effects, we increased error rates by varying a (perceived) response deadline such that all participants made between 5 and 30% errors. Thus we manipulated each participant’s overall error rate, and directly examined the switch cost, i.e., how many more errors participants made when the target level switched, compared to errors on all other trials.

Furthermore, besides again contrasting participants on creative achievement, Experiment 2 also investigated whether attentional persistence varies with divergent thinking, another measure of creativity. Previous investigations found their measure of creativity (weighted toward divergent thinking) related to attentional flexibility (Stavridou and Furnham, 1996; Vartanian, 2009). If our attention task measures the same type of attentional flexibility, high divergent thinkers in our sample should exhibit the reverse effect of high creative achievers, i.e., high divergent thinkers should exhibit less switching cost compared to low divergent thinkers. On the other hand, if our attention task is tapping into a different attention switching mechanism, we should see either no difference between high and low divergent thinkers, or a similar effect to that observed with high and low creative achievers.

### Method

#### Participants

Thirty-nine undergraduate students (24 female, average age = 20.47 years) participated for course credit or payment. They were either pre-selected based on their CAQ scores (high, medium, and low) from the Introduction to Psychology class, or recruited to participate for payment. This was again done in order to ensure a full range of creative achievement scores.

#### Materials and procedure

##### The creative achievement questionnaire

We assessed participant’s real-world creative achievements with the CAQ (Carson et al., 2005). CAQ scores ranged between 0 and 95, with a mean CAQ score of 13.85 (median = 10.00, SD = 16.09).

##### Abbreviated torrance test for adults

To examine divergent thinking, we used the abbreviated torrance test for adults (ATTA: Goff and Torrance, 2002), a shortened form of the TTCT (Torrance, 1974), a standard measure of creative potential (Kim, 2008). The ATTA consists of three activities (3 min each), one involving verbal responses (e.g., identifying problems of being able to fly without being in an airplane or a similar vehicle), and two involving figural responses (e.g., using incomplete figures to make pictures). Responses are scored for fluency (i.e., a count of the number of pertinent responses), originality (i.e., the number of responses that are unique and original), flexibility (i.e., the number of categories used), and elaboration (i.e., the extent to which responses are detailed or elaborated upon), with summary scores summed across the three activities (Goff and Torrance, 2002). We computed a single divergent thinking score based on the averaged *z*-transformed fluency, originality, flexibility, and elaboration scores.

##### Local-global task

The attributes of the stimuli (Navon, 1977) were the same as in Experiment 1. The task procedure was similar to Experiment 1 except for three changes:

(1) Participants were instructed to respond accurately, but as quickly as possible. To induce more errors, we adjusted each participant’s (perceived) response window based on his/her response times (after practice trials) and error rates (after Block 1) to ensure participants made an average of 5–30% errors (combined across switch and non-switch trials). Participants viewed the stimulus on a white background for 200 ms, then viewed a white blank screen for 500 ms, and were instructed to make their responses (i.e., identify whether there was a letter “S” or “H” present on the screen) during that time. Thus, the white blank screen provided a visible response window. The screen then turned gray for 400 ms indicating the end of the response window. Even though participants were instructed to make their responses during the white response window, we collected and analyzed responses after the response window as well (making available response times up to 1100 ms after onset of the stimulus). Participants performed 45 practice trials, and two blocks of experimental trials of 168 trials in each block.
  After practice trials, the experimenter adjusted the response window based on each participant’s response time, to induce more errors. The response window was adjusted to 150 ms less than the participant’s average response time on the practice trials. Participants then performed Block 1, after which the experimenter adjusted the response window for Block 2 based on the following criteria: if participants made fewer than 5% of errors on Block 1, the response window was adjusted by subtracting 50 ms from their average response time on Block 1. If participants made between 5 and 20% errors, the response window remained the same as on Block 1 trials. If participants made between 20 and 30% errors, 50 ms was added to the average response time. And finally, if they made over 30% errors, the experimenter added 100 ms to the response window.
  Although we varied the perceived response window to induce similar error rates for each participant, the response window durations (from stimulus onset until the onset of the gray screen) did not differ across groups, *F* < 1 (durations of 629 ms for low, 585 ms for medium, and 601 ms for high creative achievers).
(2) In order to prevent participants from knowing when switch trials would occur, blocks presented five, seven, or nine stimuli at each level, with order of block size pseudo-randomized. Because this was done in order to make the switch less predictable, and we did not have any *a priori* predictions regarding this manipulation, data were collapsed across the three block lengths.
(3) In case the fixation cross in Experiment 1 served as a local prime, in Experiment 2 we used a fixation circle (1000 ms), sized midway between the global and the local letter stimuli.

Participants used a chin rest while performing the task to ensure the distance between the participant and the computer display was the same for all participants (60 cm).

##### General intelligence

Participants provided their SAT and ACT scores, which were converted into percentiles scores for the year 2011, and used as a measure of general intelligence (*M* = 95.26, SD = 10.69).

### Results

Participants made an average of 13.56% errors (SD = 8.21); this is higher than Experiment 1 error rates because we induced more errors by limiting the (perceived) response window. The critical point is when these errors were made – on the switch trials, or on other trials within the block. Overall, participants erred more often on the trial after the switch (*M* = 21.72%, SD = 8.89) compared to all other trials (*M* = 12.26%, SD = 8.39), *t*(38) = 10.72, *p* < 0.001.

#### Creative achievement

Because participants were initially selected within high (CAQ scores 1–8, *M* = 3.86), medium (CAQ scores 9–12, *M* = 10.15), and low (CAQ scores 13–95, *M* = 29.50) creative achievement groups, and because the CAQ score ranges differed within the groups, we examined differences between the three groups. Overall, error rates did not differ between high (*M* = 13.56%, SD = 8.14), medium (*M* = 12.69%, SD = 8.10), and low (*M* = 15.29%, SD = 8.98) creative achievers, *F*(2, 38) = 0.48, *p* = 0.62. This is not surprising because we manipulated the response window to induce roughly equal overall error rates for each participant.

**Figure 1:**
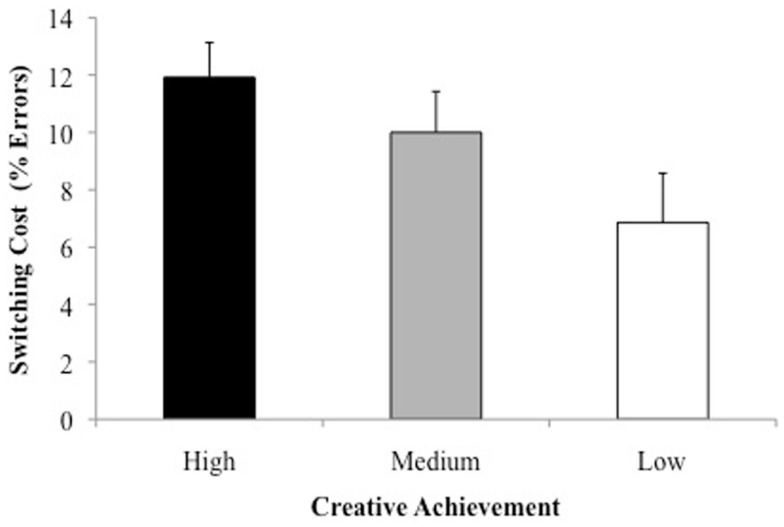
**Experiment 2: switching cost (percent errors) for high, medium, and low creative achievers**. Error bars represent standard error.

Of more interest is whether creative achievement related to switching costs. To parallel Experiment 1 analyses, and because manipulation of the response window equated overall error rates, we analyzed the switching costs, i.e., how many more errors participants of varying creative achievement made on switch trials compared to all other trials (see Figure 1). A one-way ANCOVA, controlling for general intelligence, showed the switching cost differed across the three creative achievement groups, *F*(2, 31) = 3.44, *p* < 0.05. If the data are analyzed as a mixed ANCOVA, with switch versus other trials as a factor, with general intelligence as a covariate, the interaction between group and switching remains reliable, *F*(2, 36) = 3.69, *p* = 0.04. Independent sample *t*-tests indicated that high creative achievers showed a larger switch cost (*M* = 11.92%, SD = 4.19) than did the low creative achievers (*M* = 6.86%, SD = 6.42), *F*(1, 24) = 5.59, *p* = 0.02, although the medium creative achievers (*M* = 10.00%, SD = 5.15) did not reliably differ from the other two groups, all *F*s < 2.

#### Global versus local targets

Participants erred slightly more often on global (*M* = 15.60%, SD = 9.35) than on local trials (*M* = 11.39%, SD = 9.80), *t*(38) = −2.64, *p* = 0.01. All participants erred equally often when switching from global to local targets or vice versa, and this did not interact with creative achievement (all *F*s < 1).

#### Response time results

Although errors were the primary dependent variable, we performed parallel analyses with response times as the dependent variable, excluding errors, and response times exceeding 3 SDs from the mean. Average response times (*M* = 496 ms, SD = 73 ms) were faster in Experiment 2 than in Experiment 1 precisely because we required participants to respond faster by adjusting the response window. Overall, participants showed a switch cost in response time, responding more slowly on switch trials (*M* = 509 ms, SD = 85.35) than on other trials (*M* = 490.23 ms, SD = 65.60), *t*(38) = 3.77, *p* = 0.001.

As in Experiment 1, creative achievement groups did not differ on overall response time (high CAQ *M* = 481 ms, SD = 30.86; medium CAQ *M* = 498.31, SD = 92.47, high CAQ *M* = 506.57, SD = 81.64), *F* < 1; nor did creative achievement interact with switch cost (*F* = 1.0). Thus, the greater effect for high creative achievers in error rates was not offset by a smaller effect in response time.

Participants responded more slowly on global (*M* = 513 ms, SD = 69) than on local trials (*M* = 476 ms, SD = 70), *t*(38) = −9.44, *p* < 0.001. However, the type of trial (global versus local) did not interact with creative achievement level (high, medium, and low) or switching, all *F*s < 1.7.

#### Divergent thinking

Group comparisons examining differences between high, medium, and low divergent thinkers (as measured by the ATTA) indicated that error rates did not differ between the three groups (high: *M* = 12.85%, SD = 5.41, medium: *M* = 13.00%, SD = 9.07, and low divergent thinkers: *M* = 14.85%, SD = 9.77), *F*(2, 38) = 0.23, *p* = 0.79. More importantly, the error rate switching cost did not vary by divergent thinking group, even when controlling for general intelligence (*F*s < 1). The three divergent thinking groups did not differ on overall response time, switching cost, or type of trial (global versus local), all *F*s < 1.5. Given that the divergent thinking scores were continuous, rather than falling strictly into three groups, we also performed a simultaneous multiple regression. Controlling for general intelligence, switching cost was not a strong predictor of divergent thinking, *t* = −0.93, *p* = 0.36, *b* = −0.02, SE *b* = 0.02.

### Discussion

Replicating the results of Experiment 1, when a target stimulus required participants to switch level of attention (after a block of stimuli at a consistent level), high creative achievers erred reliably more often than low creative achievers, even after accounting for general intelligence, indicating attentional perseveration to a given level of attention. However, this was not true for divergent thinking – high divergent thinkers did not reliably differ from low divergent thinkers on the number of errors they made when switching levels of attention compared to all other trials. In Experiment 2, it cannot be argued that low creative achievers were at floor, or that they were better at predicting when the switch would occur.

## Summary and Concluding Discussion

Our results demonstrate a novel link between a simple form of attention switching and creative achievement (CAQ), one measure of creativity. In contrast this switching effect did not relate to divergent thinking, another common measure of creativity. The purpose of the investigation was to examine attentional persistence in creativity. Experiment 1 found that attentional persistence, to the point of perseveration, was related to high creative achievement, even when accounting for general intelligence. Experiment 2 confirmed that attentional persistence is related to high creative achievement, and clarified that it is unrelated to divergent thinking, as measured by the ATTA.

Specifically, we examined whether people who differ in creative achievement and divergent thinking also differ in the ability to switch attention from one level (local or global) to another. Participants responded to blocks of trials in which the target stimuli occurred at one level, followed by a switch to a block at the other level. We were particularly interested in performance at the point when people had to switch between levels. In Experiment 1, participants with high creative achievements made more errors than participants with low creative achievements when switching levels, with no change in response times. High creative achievers erred at the switch because they were attending to the wrong level where the target did not occur, indicating that they have difficulty switching the focus of attention. The trouble in switching levels of attention is likely due to attentional persistence. This detriment, however, dissipated quickly: by the second trial after the switch, high creative achievers responded just as accurately as low creative achievers. Experiment 2 replicated the results of Experiment 1, confirming that high creative achievers make more errors when required to switch levels of attention, compared to low creative achievers. Divergent thinking, however, did not show this pattern – high and low divergent thinkers performed equally well.

High creative achievers exhibited cost (more errors) at the point of the switch of attention foci, but did not show benefit (such as improved response time or better accuracy) on the string of trials with targets occurring at a single level of attention. It is not likely that high creative achievers were simply trying harder on the task and paying more attention to the pattern, therefore noticing that the target level repeated for some time, because they showed no benefit in the midst of blocks. Therefore, compared to low creative achievers, high achievers inefficiently persisted at each level (i.e., such that they made errors when the level switched), without being more focused within each block.

In contrast to high creative achievers, high divergent thinkers did not show attentional persistence when switching levels of attention, suggesting that it is possible to disentangle basic underlying cognitive processes, such as capture of attention, that may differ across different components of creativity.

On the surface, the finding that high creative achievers performed worse than low creative achievers when required to switch attention seems at odds with some prior research concluding that highly creative people have greater cognitive flexibility (Martindale, 1995; Vartanian et al., 2007; Gabora, 2010). In the current results, high creative achievers were less flexible when a change in stimuli required they switch their level (global versus local) of attention.

In one study also using a global-local letter task, cognitive flexibility was inferred because highly creative people seemed to take a different approach across tasks (Vartanian et al., 2007). Specifically, high creativity scores correlated with faster responding on tasks that lacked interfering demands for attention, but also with slower responses when interference occurred within a task. For hierarchical letter stimuli, creativity correlated with slower responding to the local stimuli, for which there was a high degree of interference (from the dominant global trials), but did not relate to speed of responding on the global trials because the local stimuli did not interfere as much. Their results on the global-local task in some ways parallel ours as their more creative participants performed worse (i.e., slowed down). Vartanian et al. (2007) attributed this decrement to the effect of global precedence of their stimuli – since creative individuals have broader attention, they have difficulty ignoring the strong global stimuli, and the random order didn’t provide any cues. In our experiments, we used stimuli designed to be equally strong at the local and global levels. However, the sequence of the stimuli established a bias toward an attentional level, such that when the sequence was broken, our more creative participants also performed worse (i.e., making more errors) than less creative participants.

Still, the Vartanian et al. (2007) study and the current differ in several critical ways: the participant groups being contrasted, and the type of attentional switch necessary. Vartanian et al. (2007) contrasted groups on what they called creative potential, defined by a combination of the fluency factor on a standard divergent thinking task, an insight problem-solving task, and a self-report scale of creative personality. In order to do well on the first two measures, fast switching of attention is in itself a requirement. Our study, on the other hand, assessed creativity in terms of real-world creative achievements, as well as (in Experiment 2) divergent thinking. The second difference is that the earlier investigation used randomly ordered stimuli with an intrinsic bias toward the global level, whereas in our study the global bias was not present, and people had a chance to develop a preference for an attentional level through repetition. In addition, their creative participants slowed down (when encountering task interference), whereas our creative participants made relatively more errors (when stimuli required them to switch level of attention).

We suggest that creative people may indeed have flexible attention, perhaps in part because their primary mode of attention is defocused or global (Martindale, 1995). Simple attentional breadth on both the perceptual and conceptual levels, for example, has been suggested to foster access to remote associations and thereby facilitate creativity (Förster and Dannenberg, 2010). Similarly, decreased latent inhibition, or decreased ability to screen from conscious awareness stimuli previously experienced as irrelevant, has been associated with increased creative achievement in high-functioning people (Carson et al., 2003). Whereas this defocused attention is beneficial for detecting alternative foci (external stimuli or internal associations), it may also demand that highly creative people exert more cognitive control in order to focus even within the block, and it takes time or effort to disengage this cognitive control (see Zabelina and Robinson, 2010). Supporting this idea, high creative achievers showed a cost when switching levels of attention when a sequence of trials at one level ended, but did not show benefit (less errors or lower response times) on the trials within the sequence. In fact, high creative achievers made slightly more errors even within the blocks (in Experiment 1; Experiment 2 equated performance levels, so no difference could emerge within blocks). Therefore high creative achievers showed an increased cost when switching after each block because they had engaged cognitive control to maintain focus within the block, and needed to disengage this control at the switch. Thus, the attentional style of highly creative people may be a double edged-sword: while initially defocused attention may switch quickly, cognitive control may take longer to switch. Nonetheless, both edges of this sword have potential benefits for creativity.

Although prior studies suggested that flexible switching between global and local modes of processing may promote successful creative problem-solving (Smallwood and Schooler, 2006; Wiley and Jarosz, 2012), it appears that real-world creative acts may be at least partially dependent on increased levels of cognitive control and resulting attentional persistence. Perhaps this partially explains why artists, compared to non-artists, spend more time re-working their drawings (Kozbelt, 2008): they either have learned that to achieve anything worthwhile, they need to spend time perfecting it, or they are simply more intrinsically interested in the task. The current experiments demonstrate that the tendency for perseverance may indeed be a defining characteristic of successful creative achievement, even if it comes with the cost of perseveration in certain circumstances.

## Conflict of Interest Statement

The authors declare that the research was conducted in the absence of any commercial or financial relationships that could be construed as a potential conflict of interest.
